# A Longitudinal Investigation of Preferential Attention to Biological Motion in 2- to 24-Month-Old Infants

**DOI:** 10.1038/s41598-018-20808-0

**Published:** 2018-02-06

**Authors:** Robin Sifre, Lindsay Olson, Scott Gillespie, Ami Klin, Warren Jones, Sarah Shultz

**Affiliations:** 1 Institute of Child Development, University of Minnesota Twin Cities, Minneapolis, USA; 2 Joint Doctoral Program in Clinical Psychology, San Diego State University/University of California, San Diego, San Diego, CA USA; 3 Pediatric Biostatistics Core, Emory University School of Medicine, Atlanta, GA USA; 4 Marcus Autism Center, Children’s Healthcare of Atlanta, Atlanta, GA USA; 5 Division of Autism & Related Disabilities, Department of Pediatrics, Emory University School of Medicine, Atlanta, GA USA; 6 Center for Translational Social Neuroscience, Emory University, Atlanta, GA USA

## Abstract

Preferential attention to biological motion is an early-emerging mechanism of adaptive action that plays a critical role in social development. The present study provides a comprehensive longitudinal mapping of developmental change in preferential attention to biological motion in 116 infants at 7 longitudinal time points. Tested repeatedly from 2 until 24 months of age, results reveal that preferential attention to biological motion changes considerably during the first months of life. Previously reported preferences in both neonates and older infants are absent in the second month but do reemerge by month 3 and become increasingly pronounced during the subsequent two years. These results highlight the second month of life as a potentially critical transition period in social visual engagement.

## Introduction

Evidence from multiple species^1–7^ and multiple time points across the human lifespan^8–11^ suggests that preferential attention to biological motion (i.e., the movements of vertebrate animals) is a highly robust, evolutionarily well-conserved, and early-emerging mechanism of adaptive action. Some have called it a “perceptual life detector”^12^. A spontaneous preference for looking at biological motion is observed within the first days of life in human neonates^8,13^, and observed even upon first exposure in visually inexperienced newly-hatched chicks^1,14^. Perception of and preferential looking towards biological motion can be elicited by a variety of forms (including the motion of non-human species^8^), is remarkably robust to signal degradation^15^, and is spared after early visual deprivation, even when global motion processing is impaired^16^. Together, these features suggest a biological mechanism that is evolutionarily important and critical for survival.

In addition to the obvious immediate survival value of detecting the conspecifics upon whose care infants depend, visual preferences for biological motion play a critical role in many aspects of social and communicative development^17^. By directing their attention towards the movement of others, infants create opportunities for learning about the many social signals conveyed through biological motion. These signals include facial expressions^18^, gaze direction^19^, gestures^20^, and the intentions underlying actions^21,22^. Merely by orienting towards conspecifics, infants entreat further interaction, creating rich opportunities for learning via reciprocal social engagement^23^. The criticality of attention to biological motion in early social development is underscored by the finding that this preference is disrupted in newborns with a high familial risk for Autism Spectrum Disorder (ASD)^24^, a developmental disability characterized by social and communicative impairments, and in 24-month-old toddlers diagnosed with ASD^10,25^.

A seminal study by Simion *et al*.^8^ provided the first demonstration that human neonates display a spontaneous visual preference for point-light displays of non-conspecific upright biological motion from the very first days of life. Several studies have since replicated and extended this finding, establishing that 2- to 4-day old human neonates exhibit preferences for the motion of both human and non-human species compared to inverted, random, and rigid non-biological motion displays^13,26,27^.

Interestingly, while preferential looking towards biological motion continues into later infancy and toddlerhood^28,29^, 2-month-old infants reportedly *fail* to display such a preference^11^. Although this lack of preference is based on a single study and may initially seem surprising—given the adaptive value of preference for biological motion and its well-established presence in neonates and older infants—the timing of this *absence-in-preference* fits well within the broader literature highlighting a developmental window before and after month 2 as a period of critical transition in early infancy. Many neonatal behaviors such as reaching^30^, orienting to sounds^31,32^, and orienting to faces^7,33–39^ all show similar U-shaped developmental trajectories, with initial neonatal predispositions for engaging in these behaviors declining at around 1 to 2 months of age before reemerging thereafter.

Although the precise mechanisms underlying these transitions are unknown, one influential theoretical account posits that experience-expectant (that is, more reflex-like and largely subcortically-mediated) neonatal behaviors transition into experience-dependent ones (that is, more flexible, largely cortically-mediated, forms of behavior that build iteratively on the initial experiences afforded by neonatal predispositions)^34,36,40,41^. This theoretical account has also been advanced in the context of biological motion perception. Chang and Troje^42^ proposed that neonatal orienting towards biological motion may be driven by an evolutionarily old, experience-expectant mechanism that is sensitive to the local cues contained in biological motion. Cross-species studies support this idea, with emerging evidence demonstrating increased activity in subcortical areas (the septum and preoptic area) when newly-hatched chicks view self-propelled motion^43^ and animate motion of a live social partner^44^. This system is presumed to direct attention towards biological motion early in life, thus ensuring critical opportunities for learning that guide the development of an experience-dependent mechanism—sensitive to the global features of biological motion—that is thought to emerge in human infants between 2 and 4 months of age^42,45^. If replicated in a larger sample, an absence of preference for biological motion at 2 months would provide evidence in support of this purported transition from experience-expectant to experience-dependent mechanisms of adaptive action at around 2 months of age.

In contrast to the previous report suggesting a lack of preference at 2 months^11^, studies of infants at 3 months of age and older do provide evidence of sensitivity to biological motion by at least 3 months. Three–month-old infants can discriminate upright biological motion from inverted and scrambled displays^46^, and preferences for biological motion have been demonstrated in cross-sectional samples of 4-, 5-, 6-, and 24-month-old infants^9–11^. By at least 4 months, infants are also quite adept at extracting information conveyed by biological motion. For instance, 4- to 8-month-olds can detect invariants of actor identity and facial expression from the biological motion of faces^18^, and 6-month-olds can detect the directionality of an upright point-light walker^47^.

In addition to infants’ growing sensitivity to cues conveyed through biological motion, evidence suggests that infants’ understanding of biological motion becomes increasingly sophisticated over their first months and years. While the extent to which neonates and very young infants perceive biological motion as a socially meaningful stimulus is unknown, there is ample evidence that older infants begin to imbue point-light animations with social meaning^48^ and have clear expectations about how animate entities represented in point-light displays should behave. For instance, 5- but not 3-month-olds are sensitive to the dynamic symmetry of point-light walkers’ limbs^49^. Six- and 9-month-olds demonstrate a visual response recovery when point-light walkers violate the principle of solidity^50^ (suggesting their growing understanding that animate entities should move as cohesive global structures). And 12- but not 9-month-olds^51^ actually respond to joint attention cues produced by upright point-light animations, suggesting that 12-month-olds perceive point-light displays of biological motion as animate beings and engage with and respond to them as such^19^. Together, these studies suggest a series of important developmental changes in the perceived functional significance of biological motion: as infants gain more experience with the social world, they begin to imbue these depictions of biological motion with increasingly nuanced meaning, and subsequently engage with them in progressively different ways.

As reviewed above, the role of preferential attention to biological motion in social development has led to an extensive body of cross-sectional studies examining the manner in which infants distinguish between and preferentially attend to different forms and features of biological motion. However, no study to date has examined the development of this socially adaptive behavior in the same infants, prospectively and longitudinally, over the first months and years of life. A prospective longitudinal study is needed to assess 1) whether the reported lack of preference at 2 months—previously observed in a single study^11^ with a relatively small sample size (n = 10)—will replicate in a larger cohort of infants; 2) when the preference emerges, if indeed its absence is found at 2 months; and 3) whether the preference wanes, is sustained, or becomes more robust as infants gain increasing experience with the social world.

We sought to address these questions by measuring the longitudinal development of preferential attention to biological motion in 116 (67 male, 49 female) infants at 7 longitudinal time points between 2 and 24 months of age. These time points were densely sampled in the first five months of life (data were collected at 2, 3, 4, and 5 months), and at 9, 15, and 24 months.

**Figure 1:**
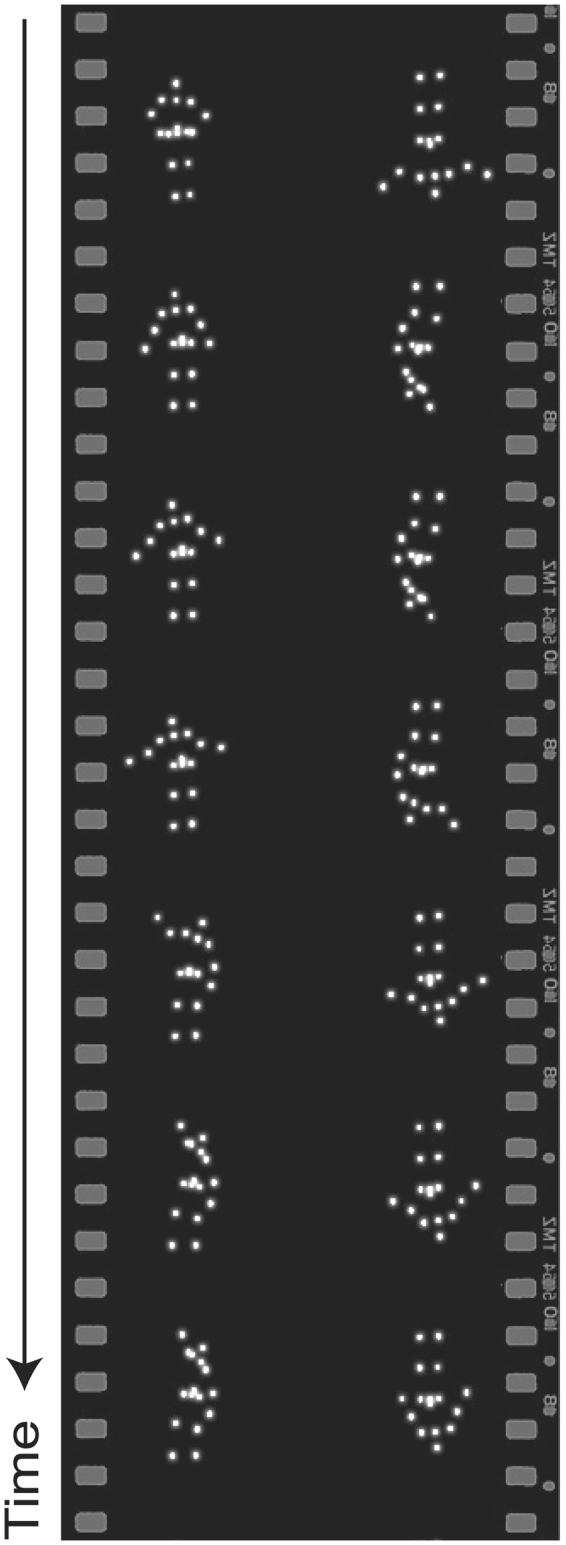
Example still images from one point-light biological motion animation. Each biological motion animation showed an upright and inverted figure and included an accompanying soundtrack that matched the actions of the upright figure. Upright and inverted figures were identical except that the inverted figure was rotated 180 degrees and was played in reverse order. The presentation of the upright figure was counterbalanced to appear on the left and right side of the screen equally often.

To measure preference for biological motion, we used five pairs of counterbalanced point-light animations, as in Klin *et al*.^10^ (see Fig. 1). The animations were created with a live actor using motion capture technology (Synertial, Brighton, UK), and consisted of the actor playing children’s games such as “Peek-a-boo” and “Pat-a-cake.” Motion capture sessions included simultaneous audio-recording in order to create multimodal stimuli that more closely approximate real-life social experiences. The experimental task was a preferential looking paradigm: on one half of the stimuli presentation screen, an upright point-light animation was shown; on the other half of the screen, an inverted (upside-down) version of the same animation was played in reverse order. Inverted presentation disrupts the perception of biological motion in young children^52^, but preserves motion complexity, speed, and gestalt coherence^10^. Only the audio soundtrack for the upright animation was played.

Consistent with past observations^53^, piloting in very young infants (2–3 months) revealed that trials shorter than 30 seconds often failed to reliably elicit interstimulus shifting (i.e. shifting fixation between stimuli presented on either side of the midline). To facilitate visual exploration of the simultaneously presented stimuli, the animations used in Klin *et al*.^10^ (having a mean duration of 30.5 s) were modified by playing two unique (*i*.*e*., not repeated) animations in succession (hereafter referred to as the Familiarization Segment and the Preference-Test Segment, respectively), resulting in a mean trial duration of 61 s (range: 57.1 to 66.3 s).

Percentage of visual fixation time to the upright and inverted animations was measured using eye-tracking technology, with data collected at 60 Hz. Linear mixed-effects regression models were used to evaluate mean trajectories of visual fixation on the upright figure during the Preference-Test Segment. In this context, “trajectories” refer to the model-based mean estimates over longitudinal time points. For each model, age (7 levels) was the only fixed effect. The random effects were the participant-level intercepts with an unstructured covariance structure, stratified for each level of age (2, 3, 4, 5, 9, 15, and 24 months). Residual errors for the modeled outcome were asymptotically normal, confirmed via histogram, boxplot, and quantile-quantile probability plot. To test whether percentage of fixation time to the upright figure differed from chance (50%) we tested the resulting regression model means using t-tests.

## Results

### Preferential attention to biological motion between 2 and 24 months of age

**Figure 2:**
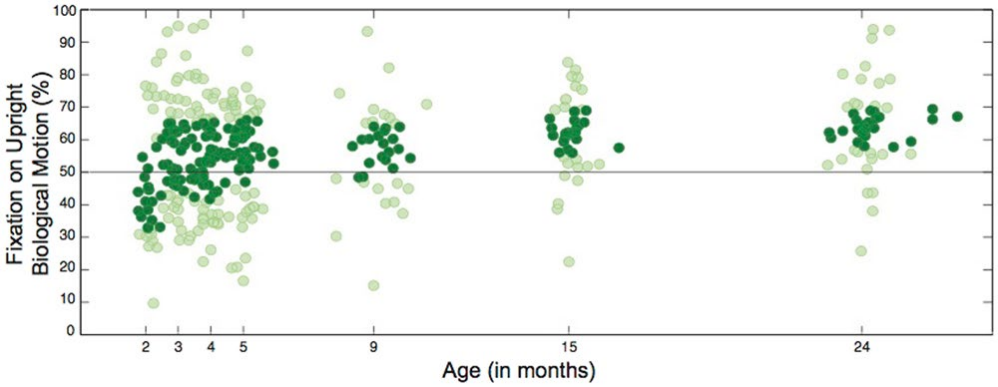
Raw data for individual data collection sessions indicating preferential attention to upright biological motion between 2 and 24 months of age. The horizontal line denotes equal looking towards upright and inverted biological motion stimuli (50%). Data are shaded to indicate distribution: darkly shaded data markers indicate the interquartile range (spanning 25^th^ to 75^th^ percentiles) while lightly shaded data markers span the minimum to maximum values. Preferential attention to upright biological motion increases with age while the interquartile range (variance) decreases.

**Figure 3:**
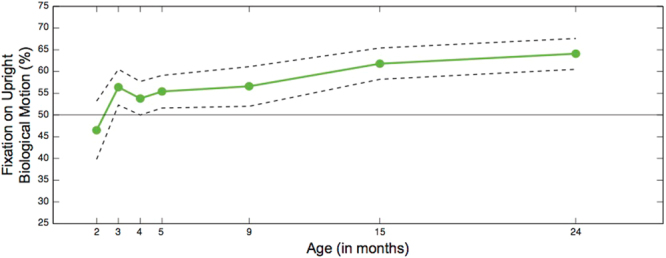
Model-based mean estimates of preferential attention to upright biological motion between 2 and 24 months of age. Dashed lines represent 95% confidence intervals. The horizontal line denotes equal looking towards upright and inverted biological motion stimuli (50%). Percentage of fixation time to upright biological motion stimuli increases with age (*F* = 5.85, *p* < 0.001). While 2-month-old infants *do not* look preferentially at the upright figure (M = 46.5%, 95% CI [39.8–53.2%], *p* = 0.299), a preference is observed by month 3 and increases through month 24.

Raw data indicating preferential attention to upright biological motion (percentage of fixation time on the upright figure) are shown in Fig. 2. Consistent with the results of Fox & McDaniel^11^, preferential attention to upright biological motion was absent at 2 months (M = 46.5%, 95% CI [39.8–53.2%], *p* = 0.299). However, results from the mixed model indicate a significant association between age and preferential attention to biological motion (*F* = 5.85, *p* < 0.001), with fixation on the upright biological motion stimuli increasing over developmental time (Fig. 3). Mean estimates from the mixed model revealed significant preferential attention to upright biological motion at 3 months (M = 56.4%, 95% CI [52.3–60.5%], *p* < 0.01), at 5 months (M = 55.4%, 95% CI [51.6–59.1%], *p* < 0.01), at 9 months (M = 56.6%, 95% CI [52.0–61.1%], *p* < 0.01), at 15 months (M = 61.8%, 95% CI [58.2–65.4%], *p* < 0.001), and at 24 months (M = 64.1%, 95% CI [60.5–67.6%], *p* < 0.001), with preference close to significance at 4 months (M = 53.8%, 95% CI [50.0–57.7%], *p* = 0.058) (all *p*-values adjusted for multiple comparisons (7 total) using the Benjamini-Hochberg method^54^, with a false discovery rate of 5%). As expected, the preference for biological motion—first observed at 3 months—increased with age, with percent change in looking time on the upright figure increasing by 13.65% between 3 and 24 months.

### Duration of viewing data collected and extent of visual exploration between 2 and 24 months of age

To examine whether the absence of preferential attention to upright biological motion observed at 2 months could be due to age-related differences in attention to task or developmental differences in visual exploration, we compared the duration of viewing data collected as well as the percentage of total time spent saccading between each age group. For duration of viewing data collected, we focused specifically on time spent maintaining stable fixation, blinking, or saccading, measured in seconds, exclusive of any time spent looking offscreen or missing data due to participant movement. For extent of visual exploration, we focused on percentage of time spent in saccadic eye movements. Linear mixed-effects models (as described above) were used to evaluate the effect of age (7 levels) on both duration of viewing data collected and on extent of visual exploration during the Preference-Test Segment. Pairwise comparisons, adjusted for multiple comparisons using Tukey’s HSD procedure, revealed that 2-month-olds had *greater* durations of total viewing data relative to 5-month-olds (M = 98.4 s, 95% CI [79.47 s-121.87 s] and M = 63.27 s, 95% CI [54.23 s-73.8 s], respectively, *p* < 0.05). Two-month-olds also showed a trend towards *more* time spent saccading relative to 5-month-olds (M = 17.7%, 95% CI [14.2%-21.3%] and M = 12.4%, 95% CI [11.4%-13.5%], respectively, *p* = 0.08). No other significant pairwise differences were observed between 2 month-olds and other age groups in amount of viewing data collected or in amount of visual exploration (all *p*’s > 0.11). Given that 2-month -olds showed similar—and, in some cases, greater—attention to task and extent of saccading relative to older age groups, results suggest that the lack of preference at 2 months is unlikely to be explained by developmental differences in attention or extent of visual exploration.

### Further Supplementary Analyses

#### Preferential Attention as a Function of Segment

While the distinction between Familiarization and Preference-Test Segments was based on initial piloting of the task, we also tested for segment effects before conducting analyses of preferential attention during the Preference-Test Segment. As expected, linear mixed-effects model results indicated a significant main effect of Segment, in which the overall mean of preferential fixation was significantly greater during the Preference-Test Segment than the Familiarization Segment (*F* = 34.8, *p* < 0.001), indicating that preferences were more clearly observed after an initial 30 s exposure had elapsed (see Supplementary Fig. S1). The interaction between Segment (Familiarization or Preference-Test) and Age (months 2, 3, 4, 5, 9, 15, and 24) as a predictor of visual preference was not significant (*F* = 0.35, *p* = 0.911), indicating that preferences were more clearly observed in the Preference-Test Segment compared to the Familiarization Segment at all ages. These results confirm expectations that preferences are more clearly observed with longer trial duration (i.e., after an initial familiarization period has elapsed). Analyses of preferential attention to biological motion across both Familiarization and Preference-Test Segments can be found in Supplementary Fig. S2.

#### Audiovisual Synchrony

To enhance naturalistic validity, the biological motion stimuli in the present study contained bimodal information: motion and sound. Synchronous co-occurrence of change in motion and change in sound, audiovisual synchrony (AVS), and cross-modal contingencies more generally, have been shown to guide visual attention in typically developing infants^55^, and may scaffold infants’ visual selective attention in social contexts^56^. To determine whether levels of AVS were responsible for viewing preferences in the present study, we conducted a series of additional analyses: while preference for upright biological motion was consistently observed at each longitudinal time point from 3 until 24 months, preferential attention was not positively associated with levels of AVS at any age (see Tables S3 and S4 as well as the *Audiovisual Synchrony* section in Supplementary Information).

## Discussion

Although it is well established that preferences for biological motion play an important role in many aspects of social and communicative development (e.g. Simion *et al*.^37^), knowledge of developmental change in such preferences has been based largely on an amalgam of cross-sectional studies using a variety of stimuli and procedures. Here we provide a longitudinal mapping of developmental change in preferential attention to biological motion by investigating preferential looking to upright biological motion at 7 longitudinal time points between 2 and 24 months of age. Our results show that preferences for upright biological motion change over the first months and years of life: 2-month-old infants do not exhibit a visual preference for biological motion, but the preference emerges by month 3 and continues to increase until the final time point of data collection at 24 months (*F* = 5.85, *p* < 0.001).

The current data indicate that 2-month-old infants *do not* show a preference for looking at upright compared with inverted biological motion displays (M = 46.5%, 95% CI [39.8–53.2%], *p* = 0.299), strengthening support for the notion that preferential attention to biological motion—reported in both neonates^8,13,26,27^ and older infants^10,11^—is absent in the second month of life. Although null results should always be interpreted with caution, three features of the present study strengthen the evidence for this lack of preference, first described by Fox & McDaniel^11^, in 2-month-old infants. First, the present study used a larger sample of 2-month-olds (n = 33) relative to that of Fox and McDaniel (n = 10), providing greater power to detect a preference, if present. Second, the current study used relatively longer trials (mean duration = 61 s), with analyses focused on the second half of the trials to allow sufficient time for interstimulus shifting and visual exploration^53^. By contrast, Fox & McDaniel^11^ used relatively short trial times (mean duration = 15 s), a procedural difference that has been cited as a possible explanation for the lack of preference observed at 2 months^8^ and a difference that is consistent with other evidence suggesting that many failures to detect visual preferences before month 4 are due to short trial durations of 30 s or less^53^. Finally, calibration accuracy (see Supplementary Fig. S3), attention to task, and amount of visual exploration were comparable between 2-month-olds and older infants, making it unlikely that the lack of preference at 2-months is attributable to any of these factors. Thus, the lack of preference at 2 months in the current study—observed even when using a larger sample size, longer trial durations, and high-quality eye-tracking data—provides convergent evidence strengthening the previous report of no preference for biological motion in 2-month-old infants^11^.

Taken together, the extant literature on biological motion perception in neonates^8,13,26,27^, and the present results, provide support for the hypothesis that a preference for biological motion may be present at birth followed by a period of decline at around 2 months of age, and then reemerge soon thereafter^42,45^. It should be noted that, guided by a methodological desire to more closely approximate multi-modal, real-life social experiences of human neonates, the present study used multimodal (i.e., audio and visual) stimuli, while previous studies examining these processes in neonates used visual-only stimuli^8,13,26,27^. Comparisons between the present results and those of previous studies must bear this in mind. However, if supported in future longitudinal studies from birth to 2 months, this pattern of results would be consistent with the notion that the second month of life marks a period of transition from experience-expectant (i.e., more ‘reflex-like’, largely subcortically-mediated responses) to experience-dependent mechanisms of adaptive action (i.e., more voluntary actions that build iteratively on initial newborn experiences and context-dependent learning)^34,36,40^. While this hypothesized transition need not imply the ‘disappearance’ of initial newborn predispositions (to the contrary, data from adults provides evidence of developmental continuity in the role of subcortical pathways for detecting conspecifics^41^), it may signal the emergence of increasingly interactive, experience-dependent ways of engaging with the world that build upon and/or modulate early newborn predispositions^57^. Future longitudinal studies of developmental change in preferences for biological motion from birth to 2 months are needed to provide definitive evidence for this purported transition and to investigate the mechanisms underlying such changes^58^.

Rather than waning or remaining stable at a fixed level, infants’ preferential attention to biological motion becomes increasingly robust from 3 until 24 months, with percentage of time spent looking towards upright biological motion increasing by 13.6% during this period. This increase suggests that infants’ growing experience with others may contribute to the strengthening of their preference, and vice versa, underscoring the tight coupling between attention to biological motion and social development in the first two years of life. While newborn predispositions for preferential attention to biological motion may serve primarily as an orienting mechanism, scaffolding social development by creating opportunities for interacting with and learning from others, preferences for biological motion in later infancy may reflect more active seeking of social information: the same stimuli acquire new meaning to the infant and are looked at in new ways. While this interpretation is consistent with previous reports^19^, future longitudinal studies are needed to more directly map the reciprocal relationship between change in infants’ visual engagement with biological motion and change in infants’ understanding of the rich social information conveyed by biological motion.

These results also have important implications for understanding atypical social development. In contrast to the typically-developing infants in the current study who showed increasing preferential attention to upright biological motion from 3 until 24 months of age, a previous study—using the same stimuli—revealed that 24-month-olds with Autism Spectrum Disorder (ASD) fail to show a preference for upright biological motion^10^. Absence of preferential looking to biological motion in toddlers with ASD suggests (a) an already-divergent course of earlier development, in which this mechanism of social adaptive action has been disrupted, and (b) a series of future developmental consequences in which learning in children with ASD is likely to proceed along an increasingly different course. The early-emergence of these preferences in typical development suggests that the onset of disruption is likely to occur very early in ASD. Ideally, the 2- to 24-month developmental trajectories of preferential attention to biological motion presented herein can provide a future benchmark against which to identify early departures from typical trajectories, and the consequences thereof, in ASD.

In addition, the hypothesis advanced herein that the second month of life marks a critical transition in engagement with biological motion highlights a specific developmental window—before and after month 2—as a potentially important target for future investigation in ASD. A recent study suggested that early transitions in another mechanism of social adaptive action—looking at the eyes of others—is similarly disrupted, beginning by at least month 2, in infants with ASD^59^. This study revealed that infants with ASD exhibited a decline in eye-looking from 2 until 24 months of age, a pattern not observed in typically-developing infants. Interestingly, however, early levels of eye-looking were *not* immediately diminished in infants later diagnosed with ASD; instead these infants exhibited a slight but statistically significant increase in eye-looking at 2 months, which then declined. By contrast, typically-developing infants exhibited a relative low point in eye-looking at 2 months, which then increased. The timing of this difference raised an intriguing hypothesis about the early pathogenesis of ASD: while experience-expectant, reflex-like orientation to eyes may initially, at least superficially, be present, a transition to learned, experience-dependent forms of eye-looking may fail^58^. Critically, however, the presence of eye-looking in ASD at 2 months does not necessarily mean that the behavior is functioning in a normative manner. Indeed, initial reflex-like orienting to social stimuli has been hypothesized to be impaired at birth^24^ in ASD.

Examining preferences for biological motion during this same developmental period can provide another avenue for testing this hypothesis about transitions from experience-expectant to experience-dependent mechanisms. Focusing on early preferences for biological motion may be especially fruitful given the established role of biological motion perception in imprinting^60,61^ as well as well-described animal models of the neural systems involved in filial orienting and attachment^62^. These models provide a natural target for investigating the neurodevelopmental changes that accompany critical behavioral transitions in early infancy and for determining how these mechanisms may be disrupted in infants with ASD.

## Methods

### Participants

The research protocol was approved as bearing no significant risk by the Human Investigations Committees at Emory University School of Medicine, and the data collected were used for research purposes only. All methods were carried out in accordance of relevant guidelines and regulations. With the written, informed consent of their parent or guardian, 116 infants (67 male, 49 female) were enrolled (see Table 1). All infants were born at full-term (mean gestational age = 39.13 weeks, SD = 1.36) and were reared in homes where English was the primary language. Infants’ parents identified them as White and non-Hispanic (80.2%), White and Hispanic (6.9%), more than one race (5.2%), Asian (3.4%), or Black (3.4%), with one participant’s demographic data missing due to early withdrawal from the study. Data were also collected on the primary caregiver’s education attainment, with 73% completing a graduate or professional degree, 24% a college degree, and 3% a high school degree.

**Table 1:** Participant Characterization Data at each Longitudinal Data Collection Session.

Session^a^	n	Males	Females	Age, in months^b^
1.5–2.4 months	33	16	17	2.13(0.19)
2.5–3.4 months	61	24	37	2.99(0.26)
3.5–4.4 months	63	23	40	3.94(0.26)
4.5–5.9 months	62	26	36	5.04(0.31)
7.0–11.0 months	39	14	25	9.19(0.61)
13.0–17.0 months	44	22	22	15.09(0.38)
21.0–27.0 months	51	21	30	24.27(0.76)

^a^Study visits at each of the 7 longitudinal time points were scheduled to fall within these age-delimited boundaries.

^b^Age (in months) is represented as mean(SD).

Participants were selected from a cohort of infants participating in a prospective longitudinal study of visual engagement in infants at low- and high-risk for ASD. Given ongoing research in infants at increased risk for neurodevelopmental disorders (e.g., Jones & Klin^59^), “typicality” in the current study was defined at enrollment by the absence of familial risk factors for neurodevelopmental disorders: All infants had no family history of ASD in first, second, or third degree relatives, no developmental delays in first degree relatives, no pre- or perinatal complications, no history of seizures, no known medical conditions or genetic disorders, and no hearing loss or visual impairment. Eye tracking data were collected at 7 longitudinal time points: months 2, 3, 4, 5, 9, 15, and 24. The average age of first visit was 2.16 months (SD = 1.36), and all infants were contacted at each subsequent longitudinal time point to participate in data collection. As is typical in infant longitudinal studies with many sampled time points, some participants missed one or more scheduled data collection visits, but were still included in analyses.

While the present study was undertaken as a study of typical infant development, data collection in infants at high familial risk for ASD is ongoing. When a sufficient number of children have reached the age of stable diagnostic ascertainment, future analyses focused on measuring developmental trajectories of preferential attention to biological motion in ASD will be presented.

### Stimuli and Experimental Paradigm

The location of the upright animation was counterbalanced to appear on the left and right side of the screen equally often, creating a total of ten video trials. To facilitate visual exploration of the simultaneously presented stimuli, the animations used in Klin *et al*.^10^ (having a mean duration of 30.5 s) were modified by playing two unique (*i*.*e*., not repeated) animations in succession (referred to as the Familiarization Segment and the Preference-Test Segment, respectively), resulting in a mean trial duration of 61 s (range: 57.1 to 66.3 s). Animations were paired pseudo-randomly, such that each of the five unique animations appeared once as a Familiarization Segment and once as a Preference-Test Segment. Necessarily, across Familiarization and Preference-Test Segments the location of the upright animation (on the left or right side of the screen) remained consistent.

Point-light biological motion animations ranged in size from 7.0**°** to 8.0**°** of horizontal visual angle and 9.0**°** to 10.0**°** of vertical visual angle. The upright and inverted animations were separated by approximately 8.0**°** to 10.0**°** of visual angle.

### Procedure

#### Experimental setting and equipment

In identical fashion to previously-published longitudinal research in 2- to 24-month-old infants^59^, two settings were used for eye-tracking data collection. One eye-tracking lab was optimized for data collection in infants aged 2 to 5 months, and the other lab was optimized for data collection from infants and toddlers aged 9 to 24 months. The primary distinction between the two settings was the use of a reclining bassinet for the younger infants, versus the use of an upright car seat for older infants and toddlers. All other aspects of the experimental procedures, including stimulus presentation, eye-tracking hardware and software, and data collection and analysis, were identical in both settings. Eye-tracking data were collected at 60 Hz using a video-based, dark pupil/corneal reflection technique with ISCAN hardware and software (Woburn, MA, USA).

#### Protocol

Infants and toddlers were accompanied at all times by a parent or caregiver. Each experimental session began with the child and caregiver entering the room while a children’s video played on the stimuli presentation screen. The experimenter buckled the child into the bassinet or car seat, standardizing each child’s eye position relative to the presentation screen (28 inches away from the screen, which subtended an approximately 24° × 32° portion of each child’s visual field). During testing, both the caregiver and experimenters were out of the child’s view but were able to monitor the child throughout the entire experiment via a live video feed.

Once the child was comfortably watching the children’s video, data collection began by pausing the video and presenting calibration targets on an otherwise blank background. A five-point calibration scheme was used, consisting of bright, animated targets, ranging in size from 1° to 1.5° of visual angle, played with accompanying sound. After the calibration routine was completed and calibration accuracy was verified, the experimental videos were shown. A “centering-stimulus” (i.e., a rotating pin-wheel with accompanying sound) was presented prior to each experimental video to attract viewer attention to the center of the screen. Calibration accuracy was checked repeatedly throughout the experimental protocol to measure any change in calibration accuracy, allowing the experimenter to pause the experiment and recalibrate if calibration accuracy drift exceeded 3**°**. Plots of fixation locations relative to calibration targets show that accuracy was well within this limit at all ages (see Supplementary Fig. S3).

Videos shown at each longitudinal time point were drawn in pseudo-random order from the pool of 10 trials to ensure some novel and some repeated trials at each data collection session. A maximum of 10 trials were shown at each session, with each of the 10 trials presented only once (see Supplementary Table S2 for a detailed report on the average number of trials collected at each data collection session).

### Analysis and Measures

Automated analysis of eye movements to identify fixations, saccades, blinks and missing/offscreen data was performed using software written in MATLAB (MathWorks, Natick, MA, USA) as in Jones & Klin^59^, Klin *et al*.^10^, and Shultz, Klin, & Jones^63^. We used a velocity-based criterion for identification of saccades and fixations (saccades were identified by velocity threshold of 30° per second^64^; blinks were identified by eyelid closure indexed by speed of change in pupil size (as the closing eyelid covers the pupil and causes more rapid change than what typically occurs during dilation and constriction); off-screen fixations (i.e., when a participant looked away from the video screen) were identified by gaze vectors beyond screen bounds; and the remaining fixational eye movements were those with fixation coordinates *within* screen bounds having velocities less than 30° per second). In the second phase of analysis, eye movements identified as fixations were coded relative to the upright and inverted animations, as in Klin *et al*.^10^.

We employed three minimum-valid-data criteria. First, data from a Segment were included if calibration was accurate, defined by error less than or equal to 3 degrees. Second, data were included if the infant fixated onscreen for at least 20% of the Segment. Third, to ensure adequate counterbalancing—an especially important consideration in early infancy when side biases can play a strong role in infants’ preferential looking^65^—participants were required, for each valid trial in which the upright figure was presented on the right side of the screen, to have an equal number of valid trials in which the upright figure was presented on the left side. In cases with more collected (for example) right-side upright trials than left-side upright trials, we performed planned *post-hoc* counterbalancing by excluding the additional right-side trial; per *a priori* protocol guidelines, we excluded the trial that contained the least amount of usable fixation data.

At 2, 3, 4, 5, 9, 15, and 24 months, respectively, data from 71.3%, 68.5%, 69.2%, 75.6%, 53.4%, 65.7%, and 91.1% of children successfully met the minimum-valid-data criteria. At 2, 3, 4, 5, 9, 15, and 24 months, respectively, an average of 4.5, 4.1, 4.3, 4.3, 4.0, 5.2, and 5.3 valid trials were collected from each child (for a detailed description of the duration and amount of data collected see Supplementary Tables S1 and S2). In cases where data collection sessions failed to meet the criteria, causes were due to fussiness, falling asleep, or calibration failure. Eye-tracking data from 33 2-month-olds, 61 3-month-olds, 63 4-month-olds, 62 5-month-olds, 39 9-month-olds, 44 15-month-olds, and 51 24-month-olds were analyzed.

Longitudinal data analysis was performed using SAS v9.4 (Cary, NC), and statistical significance was evaluated at the 0.05 significance level. Utilizing restricted maximum likelihood estimation, under a mixed model framework in SAS PROC MIXED, missing data were assumed to be at random after visual evaluation of the participation logs for patterns in attrition. With this approach, likelihood functions were derived separately for subjects missing observations versus those with complete data, and maximized together to obtain the final model parameters. This method gives unbiased estimates and standard errors, relative to other popular methods such as data imputation and complete case-only analysis^66^.

## Electronic supplementary material

Supplementary information
