# Supplementary material for: A Longitudinal Investigation of Preferential Attention to Biological Motion in 2- to 24-Month-Old Infants
Source: Sci Rep. 2018 Feb 6;8:2527. doi: 10.1038/s41598-018-20808-0 (PMC5802706; doi:10.1038/s41598-018-20808-0)
Supplement: Supplementary file 1 — Supplementary information [file 41598_2018_20808_MOESM1_ESM.docx]

**A Longitudinal Investigation of Preferential Attention to Biological Motion in 2 to 24 Month-Old Infants**

**Online Supplementary Materials**

**Robin Sifre^1^, Lindsay Olson^2^, Scott Gillespie^3^, Ami Klin^4,5,6^, Warren Jones^4,5,6^, Sarah Shultz^4,5^**

1. Institute of Child Development, University of Minnesota Twin Cities.
2. Joint Doctoral Program in Clinical Psychology, San Diego State University and University of California.
3. Pediatric Biostatistics Core, Emory University School of Medicine.
4. Marcus Autism Center, Children’s Healthcare of Atlanta.
5. Division of Autism & Related Disabilities, Department of Pediatrics, Emory University School of Medicine.
6. Center for Translational Social Neuroscience, Emory University.

**Duration and Amount of Viewing Data Collected**

*Duration of Viewing Time as Function of Segment*. A linear mixed-effects model was used to determine whether infants’ attention waned or remained sustained during the Preference-Test Segment relative to the initial Familiarization Segment. Attention during each segment was measured by the duration of viewing time (i.e., time spent fixating, blinking, or saccading) in seconds. Mean estimates of duration of viewing time during each segment are summarized in **Table S1.** Post-hoc pair-wise comparisons of the mean estimates revealed no significant differences in the duration of viewing time between Segments at 2-, 3-, 4-, or 24-months. There was a significant difference in the duration of viewing data between Segments at 5-, 9-, and 15-months, with the average duration of viewing time declining by 27.2, 7.6, and 14.4 seconds in the Preference-Test Segment, respectively. Despite this small decline in overall viewing duration, both 5-, 9- and 15-month-olds showed a significant preference for biological motion during the Preference-Test Segment (see Results, “Preferential attention to biological motion between 2 and 24 months of age”).

*Number of Valid Trials Collected at Each Longitudinal Time Point.* The mean number of valid trials (i.e., trials meeting the minimum-valid-data criteria outlined in Methods, ‘Analysis and Measures’) per infant at each longitudinal time point, and the percentage of participants at each data collection session who contributed 2, 4, 6, 8, or 10 valid trials are shown in **Table S2**.

**Preferential Attention as a Function of Segment**

While the decision to examine preferential attention during the Preference-Test Segment was based on initial piloting of the task, we also tested for Segment effects before conducting analyses of preferential attention during the Preference-Test Segment. As expected, linear mixed-effects model results indicated a significant main effect of Segment, in which the overall mean of preferential fixation was significantly greater during the Preference-Test Segment than the Familiarization Segment (*F*=34.8, p<0.001), indicating that preferences were more clearly observed after an initial 30 s exposure had elapsed (see **Fig. S1**). The interaction between Segment (Familiarization or Preference-Test) and Age (months 2, 3, 4, 5, 9, 15, and 24) as a predictor of visual preference was not significant (*F*=0.35, *p*=0.911), indicating that preferences were more clearly observed in the Preference-Test Segment compared to the Familiarization Segment at all ages. These results confirm expectations that preferences are more clearly observed with longer trial duration (i.e., after an initial familiarization period has elapsed).

**Percent Fixation on Biological Motion Across Familiarization and Preference-Test Segments**

While our primary analyses focused on preferential attention during the Preference-Test Segment, we also examined preferential attention across both the Familiarization and Preference-Test Segments. A linear mixed-effects model was used to evaluate mean trajectories of visual fixation on the upright figure collapsed across both Familiarization and Preference-Test Segments. This modeling approach was identical to that used in the analysis of the Preference-Test Segment alone, as described in the main text. The mixed-effects model revealed a significant association between age and preferential attention to upright biological motion (*F*=9.52, *p*<0.001), with fixation on the upright figure increasing over developmental time (see **Fig. S2**). Mean estimates from the mixed model revealed a significant preference for upright biological motion stimuli at 15-months (M=58.8%, 95% CI [56.0%-61.5%], *p*<0.001) and at 24-months (M=61.3%, 95% CI [58.8%-63.8%], *p*<0.001). No preference for the upright figure was observed at 2-months (M=45.8%, 95% CI [40.0%-51.5%], *p*=0.18), at 3-months (M=53.2%, 95% CI [49.9%-56.4%], *p*=0.095), at 4-months (M=50.8%, 95% CI [47.5%-54.1%], *p*=0.63), at 5-months (M=51.8%, 95% CI [49.3%-54.4%], *p*=0.18), or at 9-months (M=53.4%, 95% CI [50.0%-56.7%], *p*=0.095). All *p*-values were adjusted for multiple comparisons (7 total) using the Benjamini-Hochberg method, with a false discovery rate of 5%.

**Audiovisual Synchrony**

To determine whether sensitivity to AVS may have guided preferential attention to the upright figure, we examined the relationship between AVS and preferential attention to biological motion at each longitudinal time point. AVS in the upright and inverted figures, as defined by the synchronous occurrence of change in motion and change in sound, was quantified for each animation, as in Klin et al.^1^. First, change in motion was measured for each point-light’s trajectory over time. Next, the velocity and the magnitude of change in velocity of each point-light’s trajectory was calculated. This served as the measure of change in motion. To measure change in sound, the audio amplitude of the soundtrack (its short-term amplitude envelope) was measured and then its rate of change, the magnitude of ΔA, was calculated. The level of AVS of each point-light was then calculated as the product of change in velocity and change in sound amplitude. This measure of AVS was computed for all point-lights on the upright and inverted sides. For additional details, please see Klin et al.^1^

Next, a difference ratio of upright to inverted AVS was computed for each animation, as in Klin et al.^1^ This generated a normalized score comparable across animations, allowing us to test whether or not preferential viewing in our stimuli was related to the relative level of AVS on the upright versus inverted side. As in Klin et al.^1^, the relationship between AVS and preferential viewing (i.e., percent fixation, averaged across individuals within an age group for a given animation) was tested by linear regression. Using this approach, the correlation between a group of infants’ mean percent fixation on the upright figure and level of AVS in each clip (5 total) was examined at each age point. As in our analyses of preferential attention to biological motion, all *p*-values were adjusted for multiple comparisons (7 total) using the Benjamini-Hochberg method^2^, with a false discovery rate of 5%.

Correlations between AVS and preferential viewing for both the Familiarization and Preference-Test Segments at each longitudinal time point are shown in **Table S3**. Results revealed that preferential attention was not significantly associated with AVS at any age (all *p*’s > .29), indicating that sensitivity to AVS is unlikely to account for the preference for upright biological motion that was observed from 3 to 24 months of age.

Although this AVS analysis approach has the advantage of replicating the analyses performed in Klin et al.^1^, one potential disadvantage is that the relatively small set size (n=5, 1 group mean percent fixation on the upright figure, and one AVS score per clip) may be underpowered to detect statistically significant associations or may yield unstable correlation results^3^. To confirm these results using a larger set size, we next computed correlations between each *individual’s* percent fixation and the level of AVS in each clip. Stated differently, each participant contributed a data point for each animation viewed, so that for every age group there were N Participants multiplied by M Trials per participant [N*M] data points input into the correlation analyses. As in the previous approach, correlations between percent fixation and level of AVS were computed at each age point and adjusted for multiple comparisons (7 total) using the Benjamini-Hochberg method^2^, with a false discovery rate of 5%. Results revealed no statistically significant association between AVS and preference for the upright figure, with the exception of 3-month-olds in the Preference-Test Segment (see **Table S4**). However, it should be noted that this relationship was *negative* (i.e., 3-month-olds’ preference was *greater* in clips with *low* AVS), a result that is inconsistent with the notion that preferences for AVS may have guided increased fixation on upright biological motion.

To summarize, both group- (**Table S3**) and individual-level (**Table S4**) correlation analyses indicate that sensitivity to AVS is unlikely to account for the preferences for upright biological motion that were observed at each longitudinal time point from 3 to 24 months. Importantly however, we do not interpret these results as indicating that infants show a general lack of sensitivity to AVS. To the contrary, cross-modal contingencies have been shown to be important factors in guiding infant attention and learning^4^. Thus, rather than suggesting that infants ignore AVS, we interpret our control analyses as indicating that within this experimental context, preferential viewing patterns are more strongly influenced by social adaptive value (*i.e.*, the upright presentation of biological motion) than by AVS alone.

**The Effect of Sex Ratio on Percent Fixation on Biological Motion**

The ratio of male to female participants varied at each level of Age (see **Table 1**), with more females than males in all but two levels of Age (2 and 5 months). To determine whether the lack of preference for biological motion at 2 months could be attributed to difference in the ratio of males to females, a linear mixed-effects model with Age (7 levels) and Sex (2 levels) was used to evaluate mean trajectories of visual fixation to upright biological motion during the Preference-Test Segment. This modeling approach was identical to that used in the analysis of the Preference-Test Segment, as described in the main text. The mixed-effects model revealed a significant main effect of Sex (*F*=3.94; *p*<.05), with males showing a greater percent fixation on the upright figure. Despite the overall main effect of Sex, percent fixation on the upright figure did not differ significantly between males and females at any of the 7 longitudinal time points tested (all *p*’s > .98). Further, no interaction was observed between Age and Sex (F=.23, *p*=.96). Given the main effect of Sex, with males showing *greater* percent fixation on the upright figure, it is unlikely that the relatively higher male to female ratio at month 2 could have contributed to the lack of preference observed. Given that previous studies on biological motion perception reported no effect of sex in adults^5,6^, children^6^, or infants^7,8^, the overall main effect of sex observed here was unexpected and highlights the need to further examine sex effects in future studies of attention to biological motion.

**Calibration Accuracy**

We examined calibration accuracy from 2 until 24 months of age to determine whether age-related differences in preferential attention to biological motion could be due to age-related differences in calibration accuracy (see **Fig. S3**). At all ages, calibration accuracy was well within 3° of the target center (the maximum allowable drift), and the probability distribution of fixation locations relative to the fixation target was heavily weighted within the central 1-2°. Given that calibration accuracy was highly comparable between age groups, it is unlikely that age-related differences in preferential attention to biological motion are attributable to calibration accuracy.

**Number of Longitudinal Observations**

As is typical in infant longitudinal studies with many sampled time points, some participants missed one or more scheduled data collection visits, but were still included in analyses. To examine the potential impact of missing longitudinal data points on our findings, additional analyses were conducted using varying thresholds for the inclusion of a participant in the longitudinal analysis. Specifically, analyses were run including only those infants with usable data at 2 or more (n=98), 3 or more (n=69), and 4 or more (n=42) time points.

As in the original analyses described in the main text, a linear mixed-effects model was used to evaluate mean trajectories of visual fixation on the upright figure during the Preference-Test Segment. The mixed-effects model revealed a significant association between age and preferential attention to upright biological motion for infants with usable data at 2 or more, 3 or more, or 4 or more time points (all *p*’s<0.001, see **Table S5**), with fixation on the upright figure increasing over developmental time (see **Fig. S4**). Mean estimates from the mixed model revealed a significant preference for upright biological motion stimuli at 3-, 5-, 9-, 15-, and 24-months for infants with data at 2 or more time points (all *p*’s<0.05, see **Table S5**); at 3-, 4-, 5-, 9-, 15-, and 24-months for infants with data at 3 or more time points (all *p*’s<0.05, see **Table S5**); and at 3-, 5-, 9-, 15-, and 24-months for infants with data at 4 or more time points (all *p*’s<0.05, see **Table S5**). As in the original analyses, preference was close to significance at 4-months for infants with data at 2 or more and 4 or more time points (*p*=0.081 and *p*=0.191, respectively; see **Table S5**). Finally, all analyses revealed an absence of preferential attention to upright biological motion at 2-months (all *p’s*>0.29, see **Table S5**). All *p*-values in each analysis were adjusted for multiple comparisons (7 total) using the Benjamini-Hochberg method, with a false discovery rate of 5%.

Results of all analyses conducted using varying thresholds for the inclusion of a participant in longitudinal analyses were consistent with the original findings (including all participants, n=116), indicating that the reported findings are robust to variations in the number of longitudinal observations.

**References**

1. Klin A, Lin DJ, Gorrindo P, Ramsay G, Jones W. Two-year-olds with autism orient to non-social contingencies rather than biological motion. *Nature*. 2009;459(7244):257-261. doi:10.1038/nature07868.

2. Benjamini Y, Hochberg Y. Controlling the false discovery rate: a practical and powerful approach to multiple testing. *J R Stat Soc Ser B*. 1995:289-300.

3. Schönbrodt FD, Perugini M. At what sample size do correlations stabilize? *J Res Pers*. 2013;47(5):609-612.

4. Bahrick LE, Lickliter R, Flom R. Intersensory Redundancy Guides the Development of Selective Attention , Perception , and Cognition in Infancy. 2004;13(3).

5. Troje NF, Westhoff C. The inversion effect in biological motion perception: evidence for a “life detector”? *Curr Biol*. 2006;16(8):821-824. doi:10.1016/j.cub.2006.03.022.

6. Hadad BS, Maurer D, Lewis TL. Long trajectory for the development of sensitivity to global and biological motion. *Dev Sci*. 2011;14(6):1330-1339. doi:10.1111/j.1467-7687.2011.01078.x.

7. Kuhlmeier VA, Troje NF, Lee V. Young infants detect the direction of biological motion in point-light displays. *Infancy*. 2010;15(1):83-93. doi:10.1111/j.1532-7078.2009.00003.x.

8. Yoon JMD, Johnson SC. Biological Motion Displays Elicit Social Behavior in 12-Month-Olds. *Child Dev*. 2009;80(4):1069-1075.

**Table S1.** *Duration of Viewing Data Collected at Each Longitudinal Data Collection Session*

| **Age, rounded** | **Familiarization Segment**  **mean(95% CI)** | **Preference-Test Segment**  **mean(95% CI)** | **p-value** |
| --- | --- | --- | --- |
| 2 | 100.5(82.8-121.93) | 98.4(81.1-119.43) | 0.741 |
| 3 | 87.7(76.2-100.93) | 87.47(76-100.7) | 0.934 |
| 4 | 79.87(69.53-91.8) | 78.73(68.5-90.47) | 0.741 |
| 5 | 90.47(78.9-103.73) | 63.27(55.17-72.53) | <0.001* |
| 9 | 84.03(68.87-102.57) | 76.37(62.57-93.2) | 0.034* |
| 15 | 116.6(96.23-141.27) | 102.2(84.33-123.83) | 0.001* |
| 24 | 117.1(99.7-137.57) | 110.73(94.27-130.07) | 0.179 |

**p* < .05

*Table S1.* Model-based mean estimates of duration of usable viewing data during Familiarization and Preference-Test Segments, reported in seconds with 95% Confidence Interval. *P*-values adjusted using Benjamini-Hochberg Method. Mean duration of viewing data was significantly greater during the Familiarization Segment than the Preference-Test Segment in 5-, 9- and 15-month-old infants.

**Table S2.** *Number of Usable Trials Collected at Each Longitudinal Data Collection Session*

| **Age, rounded** | **Valid Trials^a^** | | **2 trials (%)^b^** | **4 trials**  **(%)^b^** | **6 trials**  **(%)^b^** | **8 trials**  **(%)^b^** | **10 trials**  **(%)^b^** |
| --- | --- | --- | --- | --- | --- | --- | --- |
| 2 | 4.48 | (2.78) | 30.30 | 33.33 | 21.21 | 12.12 | 3.03 |
| 3 | 4.13 | (2.52) | 36.07 | 29.51 | 27.87 | 4.92 | 1.64 |
| 4 | 4.29 | (2.76) | 33.33 | 36.51 | 15.87 | 11.11 | 3.17 |
| 5 | 4.32 | (2.80) | 30.65 | 38.71 | 16.13 | 12.90 | 1.61 |
| 9 | 3.95 | (2.66) | 43.59 | 25.64 | 20.51 | 10.26 | 0.00 |
| 15 | 5.18 | (2.83) | 25.00 | 25.00 | 20.45 | 25.00 | 4.55 |
| 24 | 5.33 | (2.83) | 23.53 | 21.57 | 23.53 | 27.45 | 3.92 |

1. Number of valid trials at each session is presented as mean(SD).
2. Proportion of infants with usable data from *n* trials is presented as a percentage of participants at each data collection session.

**Table S3.** *Relationship between Audiovisual Synchrony and Preferential Attention to Biological Motion: Group-Level Analysis*

| **a** |  | | **b** |  |
| --- | --- | --- | --- | --- |
|  | | \|  \| *Familiarization Segment* \| \| \| \| \| --- \| --- \| --- \| --- \| --- \| \| Age,  rounded \| *r* \| *R*^2^ \| *p, unadjusted* \| *p, adjusted* \| \| **2** \| 0.42 \| 0.18 \| 0.48 \| 0.62 \| \| **3** \| 0.40 \| 0.16 \| 0.5 \| 0.62 \| \| **4** \| -0.89 \| 0.80 \| 0.04 \| 0.29 \| \| **5** \| 0.38 \| 0.14 \| 0.53 \| 0.62 \| \| **9** \| 0.70 \| 0.48 \| 0.19 \| 0.51 \| \| **15** \| 0.67 \| 0.45 \| 0.22 \| 0.51 \| \| **24** \| -0.05 \| 0.002 \| 0.94 \| 0.94 \| |  | \|  \| *Preference-Test Segment* \| \| \| \| \| --- \| --- \| --- \| --- \| --- \| \| Age,  rounded \| *r* \| *R*^2^ \| *p, unadjusted* \| *p,*  *adjusted* \| \| **2** \| -0.04 \| 0.002 \| 0.94 \| 0.94 \| \| **3** \| -0.75 \| 0.557 \| 0.15 \| 0.44 \| \| **4** \| -0.06 \| 0.004 \| 0.92 \| 0.94 \| \| **5** \| 0.79 \| 0.624 \| 0.11 \| 0.44 \| \| **9** \| -0.06 \| 0.003 \| 0.93 \| 0.94 \| \| **15** \| 0.70 \| 0.488 \| 0.19 \| 0.44 \| \| **24** \| 0.19 \| 0.038 \| 0.75 \| 0.94 \| |

*Table S3*. Regression analyses were completed at each age to test the relationship between AVS and preference for the upright figure in **(a)** the Familiarization Segment and **(b)** the Preference-Test Segment. The correlation between a *group* of infants’ mean percent fixation on the upright figure and level of AVS was examined at each age point. There was no relationship between AVS and preferential viewing in either the Familiarization Segment or the Preference-Test Segment, at any age tested. All reported *p*’s are adjusted for multiple comparisons (7 total) using the Benjamini-Hochberg method.

| **a** |  | | **b** |  |
| --- | --- | --- | --- | --- |
|  | | \|  \| ***Familiarization Segment*** \| \| \| \| \| --- \| --- \| --- \| --- \| --- \| \| Age,  rounded \| *r* \| *R*^2^ \| *p, unadjusted* \| *p, adjusted* \| \| 2 \| -0.01 \| 0 \| 0.879 \| 0.879 \| \| 3 \| -0.01 \| 0 \| 0.856 \| 0.879 \| \| 4 \| -0.04 \| 0.002 \| 0.563 \| 0.879 \| \| 5 \| 0.09 \| 0.008 \| 0.223 \| 0.519 \| \| 9 \| 0.17 \| 0.028 \| 0.072 \| 0.252 \| \| 15 \| 0.2 \| 0.042 \| 0.013 \| 0.093 \| \| 24 \| -0.02 \| 0 \| 0.796 \| 0.879 \| |  | \|  \| ***Preference-Test Segment*** \| \| \| \| \| --- \| --- \| --- \| --- \| --- \| \| Age,  rounded \| *r* \| *R*^2^ \| *p, unadjusted* \| *p,*  *adjusted* \| \| 2 \| -0.03 \| 0.001 \| 0.783 \| 0.913 \| \| 3 \| -0.26 \| 0.069 \| 0.0003 \| 0.002* \| \| 4 \| -0.03 \| 0.001 \| 0.711 \| 0.913 \| \| 5 \| 0.16 \| 0.026 \| 0.032 \| 0.111 \| \| 9 \| 0 \| 0 \| 0.96 \| 0.960 \| \| 15 \| 0.03 \| 0.001 \| 0.69 \| 0.913 \| \| 24 \| -0.03 \| 0.001 \| 0.726 \| 0.913 \| |

**Table S4**. *Relationship between Audiovisual Synchrony and Preferential Attention to Biological Motion: Individual-Level Analysis*

**p*<.05

*Table S4*. Regression analyses were completed at each age to test the relationship

between AVS and preference for the upright figure in **(a)** the Familiarization Segment and **(b)** the Preference-Test Segment. The correlation between each *individual’s* percent fixation on the upright figure and level of AVS was examined at each age point. There was a significant *negative* relationship between AVS and preferential viewing in the Preference-Test Segment at 3-months. No other relationships between AVS and preferential viewing were observed. All reported *p*’s are adjusted for multiple comparisons (7 total) using the Benjamini-Hochberg method.

**Table S5.** *Number of Longitudinal Observations and Preferential Attention to Biological Motion*

| **a.** All participants (n=116 subjects, 1,059 observations). | | | | |  |  |
| --- | --- | --- | --- | --- | --- | --- |
| **Binned Age** | **Estimate (95% CI)** | **Statistic** | ***p*-value, unadjusted** | ***p*-value,**  **adjusted** | | |
| 2 Months | 46.5% (39.8% - 53.2%) | t(32)=-1.06 | 0.299 | 0.299 | | |
| 3 Months | 56.4% (52.3% - 60.5%) | t(60)=3.11 | 0.003 | 0.007 | | |
| 4 Months | 53.8% (50.0% - 57.7%) | t(62)=2.00 | 0.050 | 0.058 | | |
| 5 Months | 55.4% (51.6% - 59.1%) | t(61)=2.86 | 0.006 | 0.008 | | |
| 9 Months | 56.6% (52.0% - 61.1%) | t(38)=2.93 | 0.006 | 0.008 | | |
| 15 Months | 61.8% (58.2% - 65.4%) | t(43)=6.59 | <0.001 | <0.001 | | |
| 24 Months | 64.1% (60.5% - 67.6%) | t(50)=7.97 | <0.001 | <0.001 | | |
| *p-value* | **<0.001** | F_6,138_=5.85 |  |  | | |
|  |  |  |  |  | | |
| **b.** Participants with Two or More Data Points (n=98 subjects, 1,005 observations). | | | | | |  |
| **Binned Age** | **Estimate (95% CI)** | **Statistic** | ***p*-value, unadjusted** | ***p*-value,**  **adjusted** | | |
| 2 Months | 46.5% (39.8% - 53.2%) | t(32)=-1.06 | 0.299 | 0.299 | | |
| 3 Months | 56.2% (52.0% - 60.4%) | t(56)=2.99 | 0.004 | 0.009 | | |
| 4 Months | 53.6% (49.7% - 57.4%) | t(61)=1.85 | 0.070 | 0.081 | | |
| 5 Months | 55.0% (51.3% - 58.8%) | t(60)=2.69 | 0.009 | 0.013 | | |
| 9 Months | 56.8% (52.2% - 61.4%) | t(37)=2.98 | 0.005 | 0.009 | | |
| 15 Months | 62.2% (58.6% - 65.8%) | t(41)=6.79 | <0.001 | <0.001 | | |
| 24 Months | 63.7% (59.8% - 67.5%) | t(41)=7.17 | <0.001 | <0.001 | | |
| *p-value* | **<0.001** | F_6,131_=5.64 |  |  | | |
|  |  |  |  |  | | |
| **c.** Participants with Three or More Data Points (n=69 subjects, 831 observations). | | | | | |  |
| **Binned Age** | **Estimate (95% CI)** | **Statistic** | ***p*-value, unadjusted** | ***p*-value,**  **adjusted** | | |
| 2 Months | 47.2% (39.9% - 54.6%) | t(29)=-0.77 | 0.449 | 0.449 | | |
| 3 Months | 56.3% (51.6% - 60.9%) | t(44)=2.71 | 0.010 | 0.014 | | |
| 4 Months | 55.5% (51.4% - 59.6%) | t(52)=2.72 | 0.009 | 0.014 | | |
| 5 Months | 54.7% (50.7% - 58.8%) | t(49)=2.33 | 0.024 | 0.028 | | |
| 9 Months | 56.7% (51.7% - 61.7%) | t(34)=2.72 | 0.010 | 0.014 | | |
| 15 Months | 63.8% (60.2% - 67.4%) | t(33)=7.81 | <0.001 | <0.001 | | |
| 24 Months | 65.6% (61.6% - 69.6%) | t(29)=7.97 | <0.001 | <0.001 | | |
| *p-value* | **<0.001** | F_6,109_=6.32 |  |  | | |

| **d.** Participants with Four or More Data Points (n=42 subjects, 588 observations). | | | | |  |
| --- | --- | --- | --- | --- | --- |
| **Binned Age** | **Estimate (95% CI)** | **Statistic** | ***p*-value, unadjusted** | ***p*-value,**  **adjusted** | |
| 2 Months | 47.1% (38.7% - 55.5%) | t(24)=-0.72 | 0.477 | 0.477 | |
| 3 Months | 57.3% (51.7% - 62.8%) | t(29)=2.69 | 0.012 | 0.019 | |
| 4 Months | 54.0% (48.3% - 59.6%) | t(34)=1.42 | 0.164 | 0.191 | |
| 5 Months | 55.8% (51.3% - 60.3%) | t(31)=2.61 | 0.014 | 0.019 | |
| 9 Months | 58.0% (52.2% - 63.7%) | t(24)=2.86 | 0.009 | 0.019 | |
| 15 Months | 64.7% (61.0% - 68.4%) | t(28)=8.09 | <0.001 | <0.001 | |
| 24 Months | 65.0% (60.6% - 69.4%) | t(19)=7.11 | <0.001 | <0.001 | |
| *p-*value | **<0.001** | F_6,76.9_=5.15 |  |  | |
|  |  |  |  |  | |

*Table S5.* Model-based mean estimates of percentage of fixation time to upright biological motion during the Preference-Test Segment between 2 and 24 months. Results are provided for all participants **(a)**, and for participants with usable data at 2 or more (**b);** at 3 or more **(c)**; or at 4 or more **(d)** longitudinal time points. All reported *p*’s are adjusted for multiple comparisons (7 total) using the Benjamini-Hochberg method.

**Figure S1.** Model-based mean estimates of percentage of fixation time to upright biological motion during the Familiarization Segment (red) and Preference-Test Segment (green). The horizontal line denotes equal looking towards upright and inverted biological motion stimuli (50%). Error bars are 95% confidence intervals. Data for each segment were collected at the same ages; data are plotted at slight offsets from the actual age in order to facilitate visual comparison between Familiarization and Preference-Test Segments. Preferential attention was significantly greater during the Preference-Test versus Familiarization Segment (*F*=34.8, *p*<0.001).

**Figure S2.** Model-based mean estimates of preferential attention to biological motion across both Familiarization and Preference-Test Segments between 2 and 24 months of age. Dashed lines represent 95% confidence intervals. The horizontal line denotes equal looking towards upright and inverted biological motion stimuli (50%). Percentage of fixation time to upright biological motion stimuli increases with age (*F*=9.52, *p*<0.001). A significant preference for the upright figure is observed at 15 (M=58.8%, 95% CI [56.0%-61.5%], *p*<0.001) and 24 months (M=61.3%, 95% CI [58.8%-63.8%], *p*<0.001).

**Figure S3.** Calibration accuracy from 2 until 24 months of age. In plots in **a**, the cross marks the location of mean calibration accuracy, and the annulus marks the 95% confidence interval. Calibration accuracy was well within 3° of the target center (the maximum allowable drift) at each age. In **b**, kernel density estimates plot the distribution of fixation locations relative to fixation targets. Smoothing bandwidth for kernel density estimates was equal to 1°. The probability distribution of fixation locations relative to the fixation target was heavily weighted within the central 1-2° at all ages.

**Figure S4.** Model-based mean estimates of preferential attention to biological motion during the Preference-Test Segment between 2 and 24 months of age. Mean estimates are given for all participants (purple), and for participants with usable data at 2 or more (green), 3 or more (blue), or 4 or more (red) time points. Dashed lines represent 95% confidence intervals. The horizontal line denotes equal looking towards upright and inverted biological motion stimuli (50%).
